# Gemcitabine Resistance in Pancreatic Ductal Carcinoma Cell Lines Stems from Reprogramming of Energy Metabolism

**DOI:** 10.3390/ijms23147824

**Published:** 2022-07-15

**Authors:** Rina Fujiwara-Tani, Takamitsu Sasaki, Tadataka Takagi, Shiori Mori, Shingo Kishi, Yukiko Nishiguchi, Hitoshi Ohmori, Kiyomu Fujii, Hiroki Kuniyasu

**Affiliations:** Department of Molecular Pathology, School of Medicine, Nara Medical University, 840 Shijo-cho, Kashihara 634-8521, Nara, Japan; takamitu@fc4.so-net.ne.jp (T.S.); t.takagi@naramed-u.ac.jp (T.T.); m.0310.s.h5@gmail.com (S.M.); nmu6429@yahoo.co.jp (S.K.); yukko10219102@yahoo.co.jp (Y.N.); brahmus73@hotmail.com (H.O.); toto1999-dreamtheater2006-sms@nifty.com (K.F.)

**Keywords:** gemcitabine, pancreatic ductal carcinoma, drug resistance, energy metabolism, ROS

## Abstract

Pancreatic ductal adenocarcinoma (PDAC) is associated with poor prognosis because it is often detected at an advanced stage, and drug resistance interferes with treatment. However, the mechanism underlying drug resistance in PDAC remains unclear. Here, we investigated metabolic changes between a parental PDAC cell line and a gemcitabine (GEM)-resistant PDAC cell line. We established a GEM-resistant cell line, MIA-G, from MIA-PaCa-2 parental (MIA-P) cells using continuous therapeutic-dose GEM treatment. MIA-G cells were also more resistant to 5-fluorouracil in comparison to MIA-P cells. Metabolic flux analysis showed a higher oxygen consumption rate (OCR) in MIA-G cells than in MIA-P cells. Notably, OCR was suppressed by GEM treatment only in MIA-G cells. GEM treatment increased mitochondrial membrane potential and mitochondrial reactive oxygen species (ROS) in MIA-P cells, but not in MIA-G cells. Glutamine uptake and peroxidase levels were elevated in MIA-G cells. The antioxidants N-acetyl-L-cysteine and vitamin C increased the sensitivity to GEM in both cell lines. In MIA-G cells, the expression of the mitochondrial transcription factor A also decreased. Furthermore, rotenone reduced the sensitivity of MIA-P cells to GEM. These findings suggest that the suppression of oxidative phosphorylation contributes to GEM resistance by reducing ROS production. Our study provides a new approach for reducing GEM resistance in PDAC.

## 1. Introduction

Pancreatic ductal adenocarcinoma (PDAC) is an aggressive cancer with a poor prognosis [1,2] and is the fourth leading cause of cancer-related deaths in Japan [3]. The 5-year survival rate of PDAC is 9%, which is the lowest among all of the organ cancers [4]. PDAC is usually detected in advanced stages owing to a lack of specific symptoms and reliable biomarkers for the early stages of the disease [5]. Chemotherapy plays an important role in PDAC treatment because it is typically identified at an advanced stage [6]. However, chemotherapy options for PDAC are limited [7,8]. Gemcitabine (GEM) is one of the first-line drugs for unresectable, locally advanced PDAC or advanced cases [9]. GEM is a nucleoside analog that is mainly used to target pancreatic cancer [10]. GEM induces apoptosis by inhibiting the elongation of DNA strands and DNA synthesis [11]. However, continuous GEM administration often results in drug resistance, making it difficult to continue treatment [12].

Tsesmetzis et al. classified the cellular mechanisms underpinning drug resistance to nucleoside analogs into three categories: (1) membrane transport, (2) intracellular activation, and (3) effector function [13]. With respect to membrane transport, decreased expression of human equilibrative nucleoside transporter 1 (hENT1) reduces intracellular uptake of GEM [14]. Increased expression of the ATP-binding protein cassette transporter that excretes GEM extracellularly reduces the intracellular GEM concentration [15]. At the level of intracellular activation, upregulation of both ribonucleoside reductase subunit M (RRM) and deoxycytidine kinase (dCK) is associated with degradation of GEM [16,17]. In the third category, cancer stem cells (CSCs) survive chemotherapy and radiation by becoming quiescent [18]. CSCs have become the focus of considerable attention because they are multipotent and can acquire drug resistance [19]. Such acquired GEM resistance is associated with new traits acquired by exposure to GEM [20]. GEM resistance is often acquired relatively early after its initiation [21]. However, the molecular mechanisms underpinning GEM resistance in PDAC have not yet been fully elucidated.

Recent studies have focused on the significance of energy metabolism in mediating drug resistance in cancer cells [22]. Generally, normal cells produce ATP via oxidative phosphorylation (OXPHOS) under aerobic conditions. In contrast, cancer cells are known to use glycolysis even in the presence of sufficient oxygen, which is known as the Warburg effect [23]. Unlike differentiated cancer cells, CSC metabolism depends on OXPHOS [24]. In GEM-resistant PDAC cells, glycolysis is associated with low levels of reactive oxygen species (ROS) and induces stemness and/or epithelial–mesenchymal transition (EMT) [25]. Therefore, it is believed that CSCs with metabolic profiles that diverge from those of differentiated cancer cells are the key mediators of drug resistance. To confirm this hypothesis, we examined the mechanism of GEM resistance with a focus on the differences in energy metabolism between parental and GEM-resistant cells.

## 2. Results

### 2.1. Establishment of a Gemcitabine-Resistant Cell Line in MIA-G Cells

**Figure 1:**
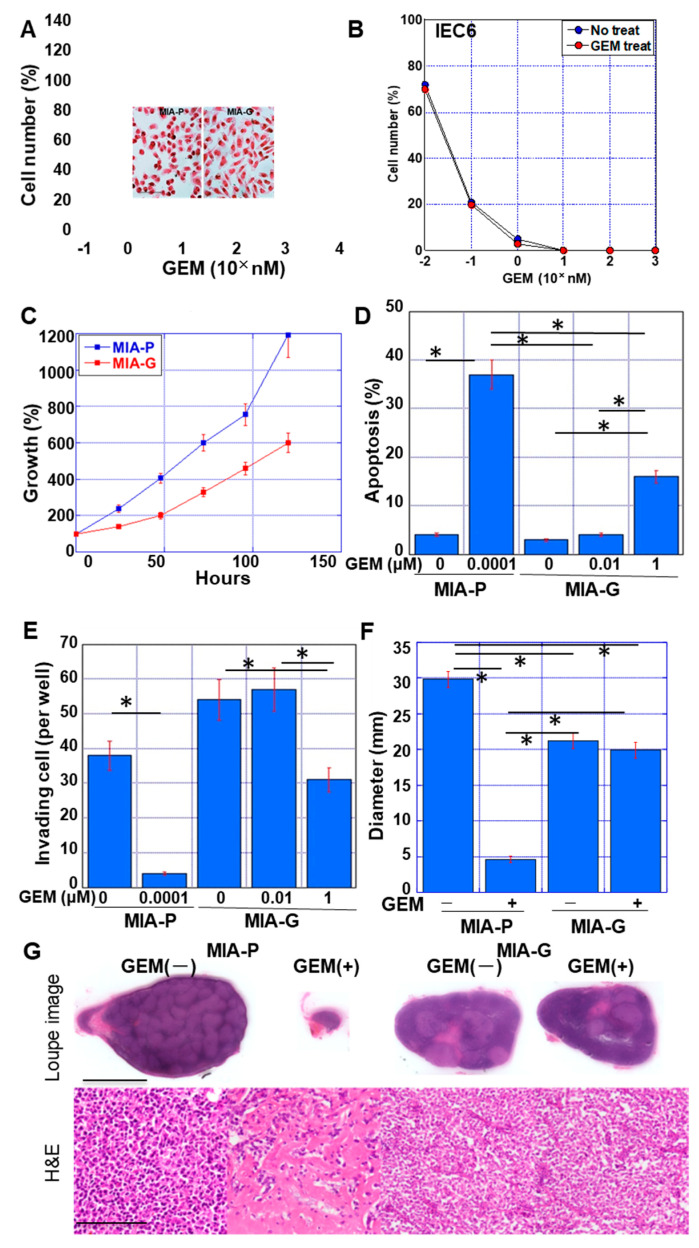
Establishment of GEM-resistant PDAC cell line. (**A**) GEM sensitivity of MIA-P and MIA-G cells treated with GEM (10^−1^ nM to 10^4^ nM). (Inset) Morphology of MIA-P and MIA-G cells without GEM. Scale bar, 50 μm. (**B**) Effect of persistent GEM (0.1 nM) treatment on GEM sensitivity in IEC6 intestinal epithelial cells. (**C**) Comparison of growth between MIA-P and MIA-G cells without GEM treatment. (**D**) Apoptosis of MIA-P and MIA-G cells upon GEM treatment. (**E**) Invasion ability of MIA-P and MIA-G cells upon GEM treatment. (**F**) The tumor diameter (mm) of MIA-P and MIA-G cells in nude mice at 6 weeks. GEM (10 mg/kg body weight) was injected into the peritoneal cavity on day 1. (**G**) Loupe image and histology of the representative tumors. Scale bar, 1 cm in loupe image and 100 μm in histology. Error bars indicate standard deviations from three independent experiments or three mice. * *p* < 0.01. GEM—gemcitabine; MIA-P—MIA-PaCa-2; MIA-G—MIA-PaCa-GEM; H&E—hematoxylin and eosin staining.

We established a GEM-resistant pancreatic cancer cell line, MIA-G, from MIA-P cells by continuous treatment with a therapeutic dose of GEM (0.1 nM) for 50 passages. MIA-G cells exhibited increased GEM resistance compared with MIA-P cells (Figure 1A). Cell morphology was altered from polygonal in MIA-P cells to short spindles in MIA-G cells, suggesting an EMT-like phenotype. In contrast to MIA-PaCa cells, the IEC6 rat intestinal epithelial cell line showed no alteration in GEM sensitivity after 50 passages of GEM (0.1 nM) treatment. The IC_50_ of GEM in MIA-G cells was 1243 ± 987 nM, whereas that of the MIA-P cells was 0.32 ± 0.03 nM. In the absence of GEM, the proliferation of MIA-G cells was slower than that of MIA-P cells (Figure 1C). GEM-induced apoptosis was also lower in MIA-G cells than in MIA-P cells (Figure 1D). MIA-G cells displayed an enhanced capacity for invasion compared with MIA-P cells in the absence of GEM (Figure 1E), and this increase in invasive ability was retained under GEM(+) conditions. Furthermore, the resistance of MIA-G cells to GEM was maintained in a mouse subcutaneous tumor model (Figure 1F). By histological examination, MIA-P cells showed decreased cell density and fibrotic change by GEM treatment. In contrast, MIA-G cells showed no histological alteration by GEM treatment (Figure 1G).

### 2.2. Resistance of MIA-G Cells to Other Anticancer Drugs

**Figure 2:**
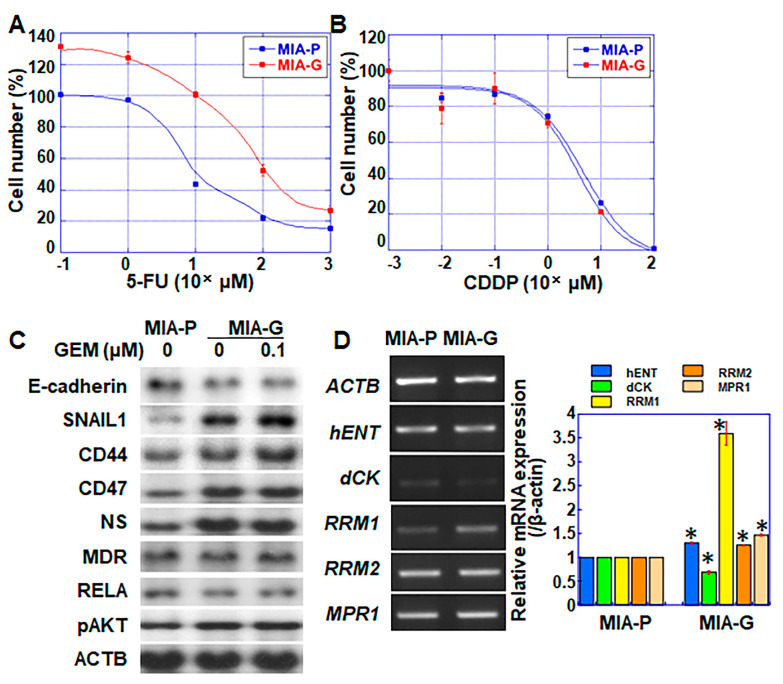
Expression of genes related to stemness and drug resistance in MIA-G cells. (**A**,**B**) Sensitivity to 5-FU and CDDP in MIA-P and MIA-G cells. (**C**) Protein levels of factors associated with EMT, stemness, multidrug resistance, and AKT signaling. In MIA-P cells, protein could not be extracted by GEM treatment due to cell degradation and death. (**D**) mRNA expression of genes associated with drug resistance. Error bars represent standard deviations from three independent evaluations. * *p* < 0.05. MIA-P—MIA-PaCa-2; MIA-G—MIA-PaCa-GEM; 5-FU—5-fluorouracil; CDDP—cisplatin; EMT—epithelial–mesenchymal transition; AKT—AKT serine/threonine kinase; NS—nucleostemin; MDR—multidrug resistance protein 1; RELA—nuclear factor kappa-light-chain-enhancer of activated B cells 3; ACTB—β-actin; hENT—human equilibrative nucleoside transporter 1; dCK—deoxycytidine kinase; RRM—ribonucleotide reductase; MRP1—multidrug resistance-associated protein 1.

Next, we examined the sensitivity of MIA-G cells to other anticancer drugs (Figure 2). MIA-G cells were resistant to 5-fluorouracil (5-FU) (Figure 2A), displaying an IC_50_ of 43.8 ± 16.7 μM, whereas that of MIA-P cells was 6.13 ± 0.38 μM. In contrast, there was no significant difference in the sensitivity to cisplatin (CDDP) between MIA-P and MIA-G cells (IC_50_ values of 4.64 ± 2.40 and 3.60 ± 2.33 μM in MIA-P and MIA-G cells, respectively) (Figure 2B).

### 2.3. Expression of Genes Associated with Stemness and Drug Resistance in MIA-G Cells

Expression of the epithelial marker E-cadherin was downregulated, whereas stem cell markers (CD44, CD47, and nucleostemin (NS)) and phosphorylated AKT were upregulated in MIA-G cells, with or without GEM treatment, compared to MIA-P cells (Figure 2C). The levels of multidrug resistance protein (MDR) were similar in both cell types. The gene expression of *dCK* was decreased and that of *hENT*, *RRM1*, *RRM2*, and multidrug resistance-associated protein 1 (*MPR1*) was elevated in MIA-G cells compared with MIA-P cells (Figure 2D).

### 2.4. Effect of GEM on Mitochondrial Status in MIA-P and MIA-G Cells

**Figure 3:**
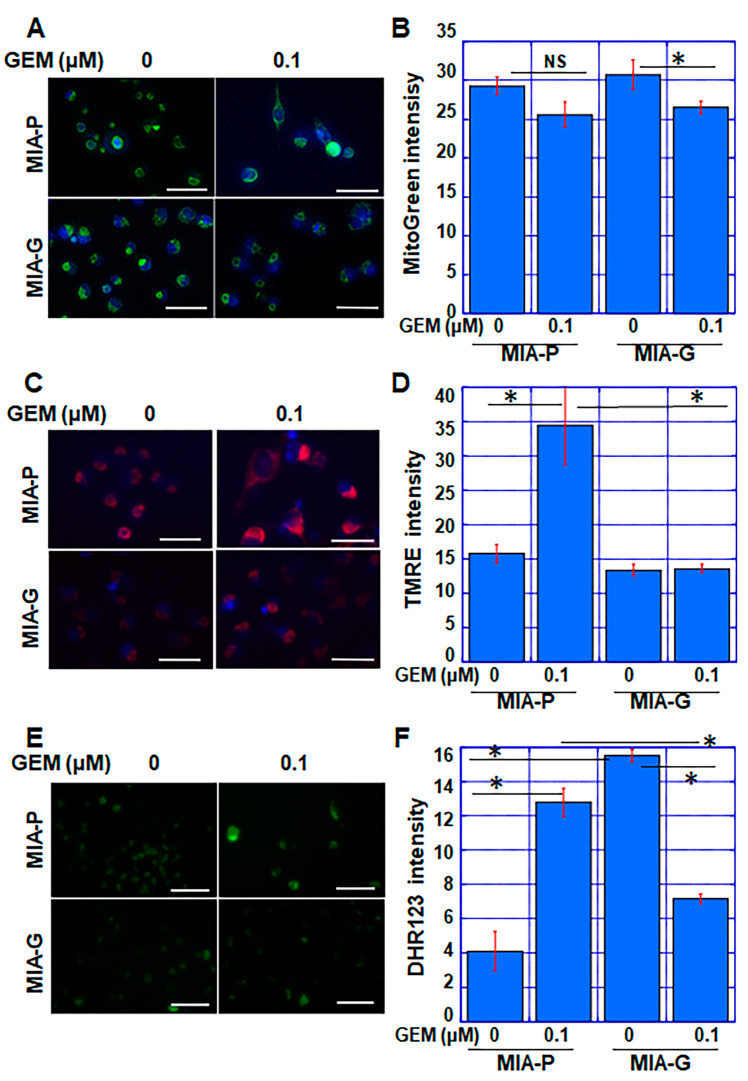
Alteration of mitochondrial status in MIA-G cells treated with GEM. (**A**,**B**) Mitochondrial volume was assessed using MitoGreen staining. (**C**,**D**) Mitochondrial membrane potential was assessed using TMRE staining. (**E**,**F**) Mitochondrial ROS production was assessed using DHR123 staining. Scale bar: 50 μm. Fluorescence intensity was assessed by measuring the average value across 20 cells. Error bars represent standard deviations from three independent evaluations. * *p* < 0.05. GEM—gemcitabine; MIA-P—MIA-PaCa-2; MIA-G—MIA-PaCa-GEM; TMRE—tetramethylrhodamine ethyl ester; DHR—dihydrorhodamine; ROS—reactive oxygen species; NS, not significant.

To evaluate the mitochondrial status after GEM treatment, we examined mitochondrial volume (via MitoGreen), mitochondrial membrane potential (MMP) (via TMRE), and mitochondrial reactive oxygen species (mtROS) (via DHR123) in both cell types (Figure 3). Although the mitochondrial volume decreased significantly in GEM-treated MIA-G cells, this change was only marginal in MIA-P cells (Figure 3A,B). When cells were treated with GEM, MMP was elevated in MIA-P cells, but not in MIA-G cells (Figure 3C,D). mtROS levels were increased by GEM treatment in MIA-P cells, but decreased in MIA-G cells (Figure 3E,F). These findings suggest that suppression of OXPHOS in MIA-G cells may decrease ROS production by GEM, which might reduce GEM-mediated cell death.

### 2.5. Differential Energy Metabolism between MIA-P and MIA-G Cells

**Figure 4:**
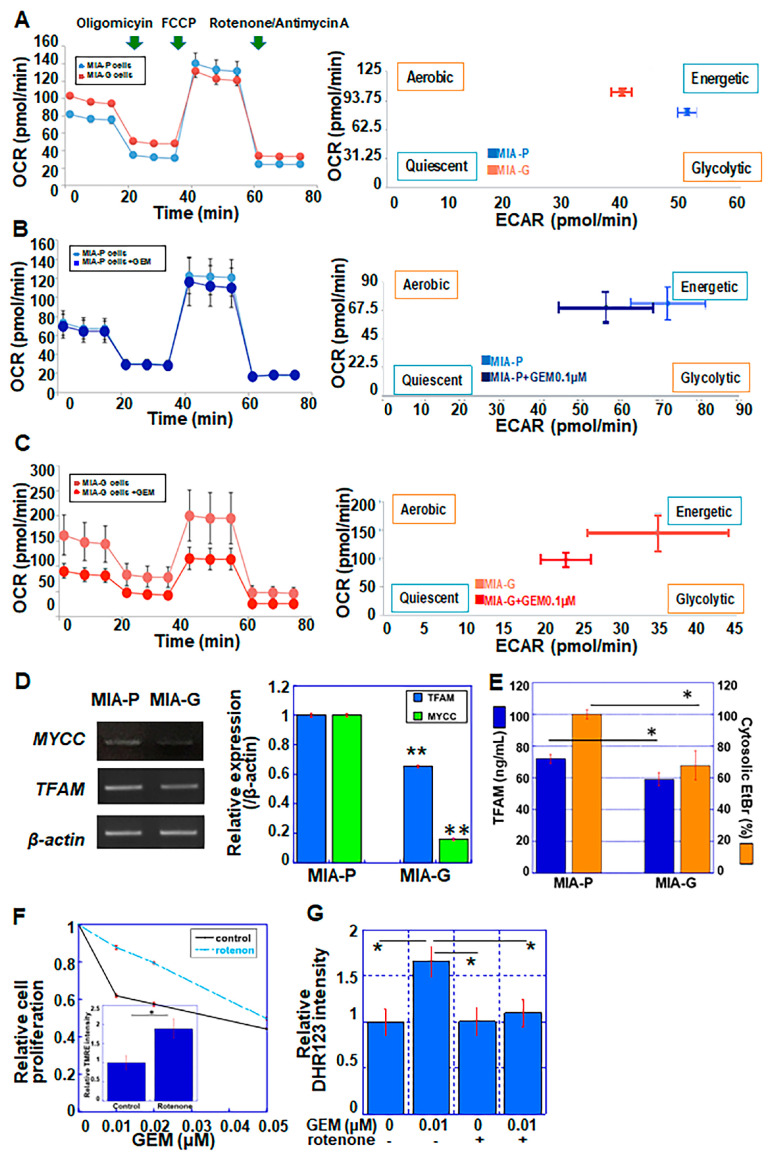
Mitochondrial respiration and metabolic profile of MIA-P and MIA-G cells. In flux analysis, 1.5 × 104 cells were sequentially treated with oligomycin (2 μM), FCCP (1 μM) and rotenone/antimycin A (0.5 μM). (**A**) MIA-P and MIA-G cells under normal conditions. (**B**,**C**) Effect of GEM (0.1 μM) on mitochondrial respiration in MIA-P (**B**) and MIA-G (**C**) cells. (**D**) mRNA expression of MYCC and TFAM. (**E**) TFAM protein levels and cytosolic EtBr intensity (%) in MIA-P and MIA-G cells. (**F**) TMRE staining and GEM sensitivity in MIA-P cells with or without rotenone (0.002 μM). (**G**) Mitochondrial ROS production was assessed by DHR123 staining in MIA-P cells. Error bars represent the standard deviation from three independent evaluations. * *p* < 0.05, ** *p* < 0.01. MIA-P—MIA-PaCa-2; MIA-G—MIA-PaCa-GEM; OCR—oxygen consumption rate; ECAR—extracellular acidification; FCCP—4-trifluoro-methoxy-phenyl-hydrazone; GEM—gemcitabine; TFAM—mitochondrial transcription factor A; DHR—dihydrorhodamine; TMRE—tetramethylrhodamine ethyl ester; EtBr—ethidium bromide.

GEM treatment generates ROS in pancreatic cancer cells [26]. As mitochondria are responsible for ROS generation [27], we evaluated the mitochondrial energy metabolism of MIA-G and MIA-P cells using flux analysis (Figure 4A–C). As shown in Figure 4A, MIA-G cells demonstrated a low spare respiratory capacity, regardless of a high basal oxygen consumption rate (OCR), compared to MIA-P cells. In MIA-P cells, the OCR was retained after GEM treatment (Figure 4B). In contrast, MIA-G cells showed markedly suppressed mitochondrial respiration following GEM treatment (Figure 4C). After GEM treatment, extracellular acidification (ECAR) decreased in both cell types; however, MIA-G cells (which displayed concurrent reduction in OCR and ECAR) entered an induced quiescent state. The expression of c-myc, which is a key factor in glycolytic energy production, was repressed in MIA-G cells compared to MIA-P cells (Figure 4D).

Mitochondrial transcription factor A (TFAM) maintains the integrity of mitochondrial DNA (mtDNA) and is associated with the mtDNA copy number [28]. Both mRNA and protein levels of TFAM were lower in MIA-G compared with MIA-P cells (Figure 4D,E). These alterations suggest that MIA-G cells may exhibit mitochondrial dysfunction, which results in suppression of OXPHOS upon GEM treatment.

Next, we investigated the effect of mitochondrial inhibition on GEM resistance in MIA-P cells. Rotenone is an inhibitor of mitochondrial respiratory chain complex I [29]. In MIA-P cells, rotenone treatment (0.002 μM) decreased the sensitivity of these cells to GEM (Figure 4F) and suppressed the levels of mitochondrial ROS generated by GEM treatment (Figure 4G).

### 2.6. Antioxidant System in MIA-G Cells

**Figure 5:**
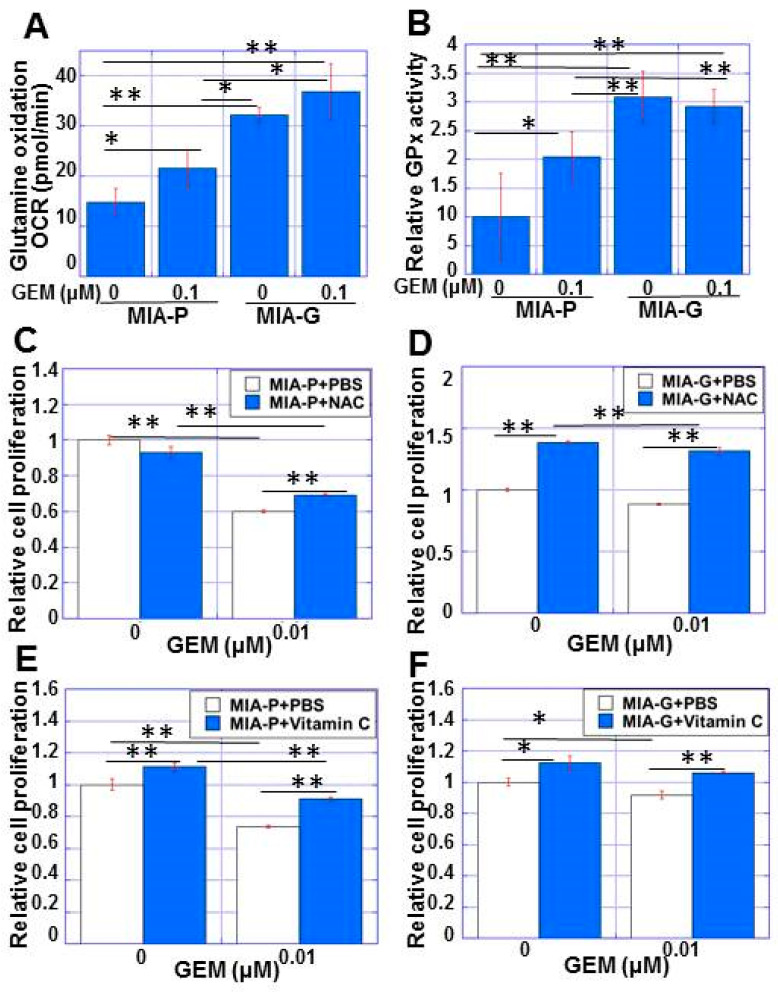
Antioxidant status of MIA-G cells. (**A**,**B**) L-glutamine uptake (**A**) and GPx activity (**B**) in MIA-P and MIA-G cells. (**C**,**D**) Effect of NAC (5 mM) on cell growth with and without GEM treatment in MIA-P cells (**C**) and MIA-G cells (**D**). (**E**,**F**) Effect of vitamin C pretreatment (1 mM) on cell growth with and without GEM treatment in MIA-P cells (**E**) and MIA-G cells (**F**). * *p* < 0.05, ** *p* < 0.01. Error bars represent standard error from three independent evaluations. MIA-P—MIA-PaCa-2; MIA-G—MIA-PaCa-GEM; GPx—glutathione peroxidase; NAC—N-acetyl-L-cysteine; GEM—gemcitabine; PBS—phosphate-buffered saline.

Next, we examined the redox status of MIA-G cells by measuring glutamine uptake and glutathione peroxidase (GPx) activity, which contribute to antioxidant defense [30]. MIA-G cells showed a higher uptake of L-glutamine and higher GPx activity than MIA-P cells, with or without GEM treatment (Figure 5A,B). GEM treatment induced L-glutamine uptake and GPx activity in MIA-P cells, although at lower levels compared with MIA-G cells. These data suggest that GPx activity may be increased in MIA-G cells to protect against mitochondrial ROS, with or without GEM.

To confirm whether reduction of ROS might induce GEM resistance, we examined the effect of the antioxidants N-acetyl cysteine (NAC) and vitamin C on GEM sensitivity (Figure 5C–F). NAC treatment slightly rescued the proliferation of MIA-P cells under GEM(+) conditions (Figure 5C). In contrast, MIA-G cell proliferation was markedly rescued with or without GEM (Figure 5D). Vitamin C treatment rescued cell proliferation with or without GEM in both cell lines (Figure 5E,F). These data suggest that the reduction of ROS is responsible for the drug resistance to GEM.

### 2.7. Mitochondrial Damage and Alteration of Energy Metabolism in Other PDAC Cell Lines

**Figure 6:**
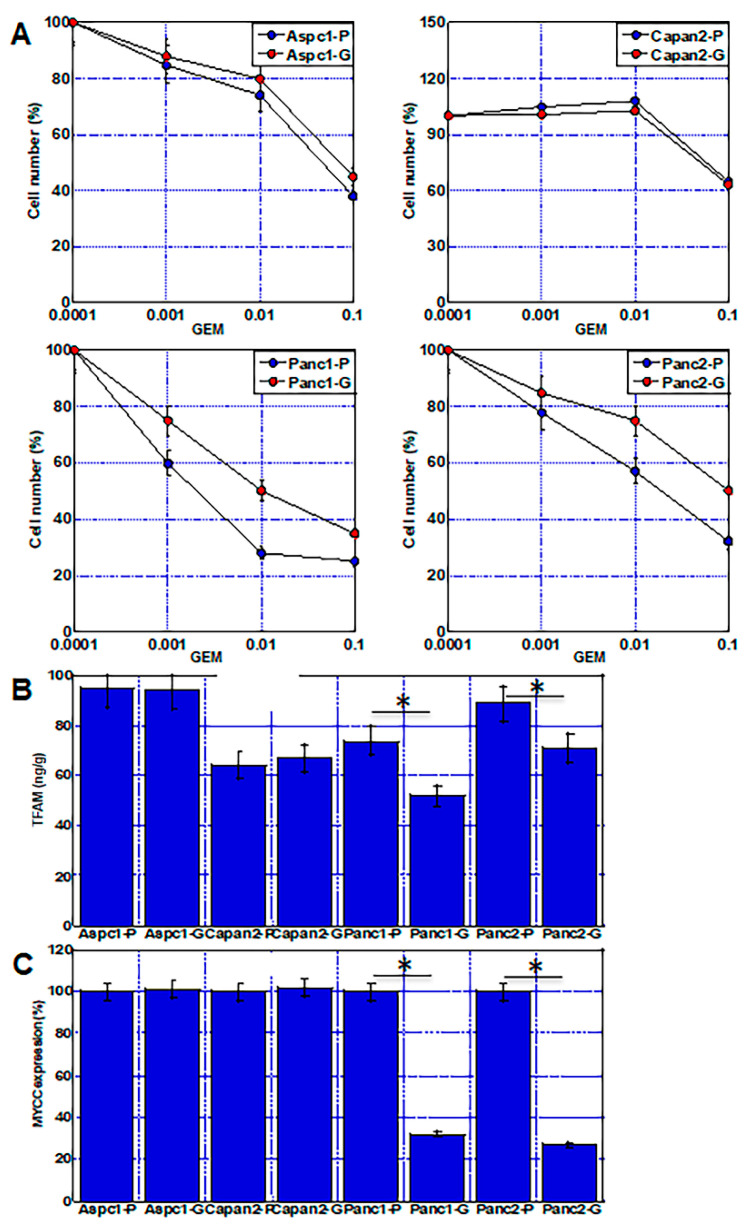
Acquisition of GEM resistance in four other PDAC cell lines. Aspc1, Capan2, Panc1, and Panc2 human PDAC cell lines were continuously treated by GEM (0.1 nM) for 50 passages. (**A**) GEM sensitivity in four PDAC cell lines. Note, Panc1 and Panc2 cells acquired GEM resistance. (**B**,**C**) TFAM protein levels (**B**) and MYCC mRNA expression (**C**) were compared between parental cells and GEM-treated cells. Error bars represent standard error from three independent evaluations. * *p* < 0.05. PDAC—pancreatic ductal adenocarcinoma; GEM—gemcitabine; Aspc1-P—parental Aspc1; Aspc1-G—GEM-treated Aspc1; Capan2-P—parental Capan2; Capan2-G—GEM-treated Capan2; Panc1-P—parental Panc1; Panc1-G—GEM-treated Panc1; Panc2-P—parental Panc2; Panc2-G—GEM-treated Panc2; TFAM—mitochondrial transcription factor A.

Four human pancreatic ductal carcinoma cell lines were treated with continuous GEM to confirm the hypothesis about GEM resistance in this study (Figure 6). Two of the cell lines (Aspc1 and Capan2) did not induce GEM resistance, whereas the other two cell lines (Panc1 and Panc2) acquired GEM resistance (Figure 6A). The GEM-resistant cell lines showed decreased expression of TFAM protein and MYCC gene expression levels, as well as of MIA-G cells (Figure 6B,C). In contrast, no such changes were observed in the two cell lines that did not acquire resistance.

### 2.8. Mitochondrial Damage and Alteration of Energy Metabolism in Human PDAC Cases

**Figure 7:**
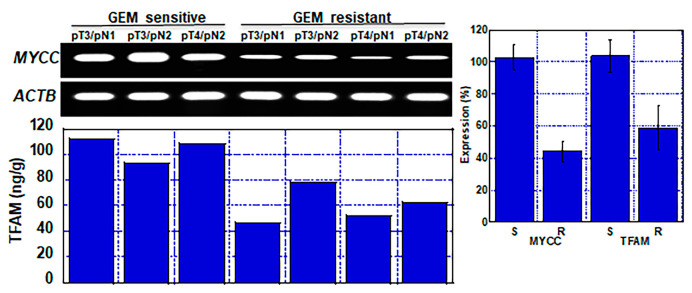
Mitochondrial damage and alteration of energy metabolism in GEM-resistant human PDAC cases. MYCC mRNA expression and TFAM protein levels were examined in three GEM-sensitive and four GEM-resistant cases. Error bars represent standard error from patients. GEM—gemcitabine; TFAM—mitochondrial transcription factor A; ACTB—beta-actin; S—GEM sensitive; R—GEM resistant.

Finally, we examined whether the mechanism by which GEM resistance is acquired through alterations in energy metabolism as revealed in the cell lines is also observed in human PDAC cases (Figure 7). MYCC gene expression and TFAM protein levels were compared between three GEM-sensitive cases and four GEM-resistant cases. The expression levels of MYCC and TFAM were lower in the resistant cases than in the sensitive cases.

## 3. Discussion

In this study, the alteration of energy metabolism by GEM exposure played an important role in the acquisition of GEM resistance by the PDAC cell line MIA-G. OXPHOS levels were higher in MIA-G cells compared with MIA-P cells under GEM(−) conditions, whereas OXPHOS was suppressed under GEM(+) conditions alongside a decrease in mtROS. In contrast, when MIA-P cells were exposed to GEM, OXPHOS was maintained, which resulted in elevation of MMP and mtROS production. These findings suggest that the reduction in OXPHOS downregulates ROS production and leads to the acquisition of GEM resistance in MIA-G cells.

In MIA-G cells, protein expression of EMT- and stemness-related genes was upregulated upon exposure to GEM. Drug resistance is considered to be a stem cell property of cancer cells [31]. Our data suggest that the reprogramming of energy metabolism and low ROS production in MIA-G cells might be associated with cancer stemness and EMT. Indeed, glycolysis-dominant energy metabolism supports low levels of ROS, which induces stemness and GEM resistance [25]. In contrast, in cancer cell lines that are dependent on OXPHOS, inhibition of OXPHOS induces apoptosis and promotes sensitivity to anticancer drugs [32].

In our study, the expression of TFAM was decreased in MIA-G cells. TFAM binds to mtDNA and contributes to the successful transcription of mitochondrial genes that encode proteins in the electron transport chain (ETC) [33]. Furthermore, TFAM contributes to the maintenance and symmetric segregation of mtDNA [28,34]. Thus, the reduction in TFAM expression suggests the presence of mtDNA damage in MIA-G cells [35,36,37]. It was previously reported that anticancer drugs impair mtDNA; for example, doxorubicin was shown to induce mtDNA damage in HeLa cells [38], and short-term GEM treatment decreased mtDNA in the pancreatic insulinoma cell line INS-1 [39]. Downregulation of mtDNA suppresses mitochondrial function and results in tumor immortalization, disease progression, and increased cancer cell stemness [40,41]. Our data indicate that continuous low-dose GEM treatment induced partial mitochondrial dysfunction by suppressing mtDNA, as suggested by the decrease in TFAM. Furthermore, GEM-treated MIA-G cells exhibited reduced mitochondrial respiration, suppressed ROS production, and subsequent GEM resistance. In contrast, the GEM-sensitive parent cell line MIA-P showed no decrease in TFAM expression and maintained OXPHOS and ROS production, with or without GEM. Cells that lack mtDNA and depend on glycolysis for energy production are known as ρ0 cells [42]. In our study, MIA-G cells did not demonstrate any mtDNA deficiencies; however, they may possess restricted mtDNA function, which might result in the reduction of OXPHOS and low ROS production, as shown by the “partial ρ0 phenotype”.

The cell-damaging effect of GEM was reduced by rotenone treatment, which decreased GEM-induced ROS production. Previous reports have shown that FCCP (4-trifluoro-methoxy-phenyl-hydrazone), which is a known uncoupler of ETC, inhibits GEM-induced ROS production and apoptosis [43]. Rotenone inhibits OXPHOS by inhibiting complex I [44] and the inhibition of complexes I and III has been shown to contribute to ROS production [45]. A combination treatment with a complex I inhibitor and GEM displayed a synergistic effect in PDAC cells with high OXPHOS [46]. In the present study, low concentrations of rotenone did not increase ROS production or reduce cell growth inhibition by GEM. Our data suggest that the prevention of OXPHOS-derived ROS production in response to GEM treatment is crucial for the acquisition of GEM resistance.

Studies on the mechanisms underpinning GEM resistance have identified some resistance-related genes [47]. In the present study, our analyses revealed a decrease in dCK gene expression and increased levels of hENT, RRM1, and MRP1 in MIA-G cells. High expression of hENT promotes GEM uptake into the cytoplasm and promotes GEM sensitivity [48]. As dCK is known to monophosphorylate pyrimidine and purine nucleoside analogs, the downregulation of dCK leads to GEM resistance [49]. RRM reduces nucleoside diphosphates (NDPs) to deoxy-NDPs (dNDPs), and overexpression of RRM contributes to GEM resistance [13]. 5-FU is also a nucleoside analog (utidine derivative) and RRM overexpression induces resistance to 5-FU [50]. From the perspective of intracellular drug metabolism, alterations in dCK and RRM might be related more broadly to drug resistance, not only to GEM but to 5-FU as well. Our finding that MIA-G cells exhibited resistance to GEM and 5-FU, but not CDDP, suggests the involvement of the dCK and RRM genes in acquiring GEM resistance in MIA-G cells. In contrast, our data indicating that GEM resistance in MIA-G cells was mitigated by antioxidants, as well as the fact that suppressing OXPHOS conferred GEM resistance on MIA-P cells, suggest that energy metabolism is significantly involved in drug resistance in cancer cells.

There is some evidence in the literature that genes involved in drug resistance may be implicated in ROS production or energy metabolism. Although there are no reports thus far indicating that RRM expression is altered by these processes, MRP1 has been shown to act on the efflux of drugs and intracellular glutathione, which maintains the glutathione disulfide (GSSG)/glutathione-SH (GSH) ratio [51,52]. In this study, the elevated expression of MRP1 in MIA-G cells may have suppressed ROS production following GEM treatment. In contrast, dCK downregulates antioxidant-associated genes via the NF-E2-related factor (NRF2)/antioxidant responsive element axis and inhibits ROS production [53]. Although dCK is a nucleoside kinase present in the cytoplasm that maintains the dNTP pool, it plays a role in supplying mitochondria with substrates for the biosynthesis of mtDNA [54,55]. Our data suggest that the downregulation of dCK might be associated with the impairment of mitochondrial function.

In this study, L-glutamine uptake and GPx activity increased in MIA-G cells. L-glutamine is imported intracellularly for conversion into GSH, whereas GPx is an antioxidant enzyme that reduces ROS by converting GSH to GSSG [30]. GPx-1 also regulates mitochondrial function and lowers mitochondrial ROS levels [56]. Thus, MIA-G cells appear to have more effective antioxidant systems than MIA-P cells. When both cell lines were treated with the antioxidants NAC and vitamin C, MIA-G cell proliferation was partially rescued. Vitamin C is a reducing agent, whereas NAC scavenges ROS and promotes glutathione biosynthesis [57,58]. Vitamin C has antioxidant properties, and it has been reported to enhance the toxicity of anticancer drugs in cancer cells by promoting the Fenton reaction [59]. Furthermore, for vitamin C to contribute to mitochondrial protection, it must be taken up by the mitochondria in its oxidized form [60]. Despite high ROS levels in MIA-P cells, the rescue effect of NAC in MIA-P cells was lower than that observed in MIA-G cells. These findings suggest that cell survival is more strongly dependent on the enhanced redox system in MIA-G cells than it is in MIA-P cells.

Our study suggests that mitochondrial damage by GEM treatment may alter energy metabolism, and subsequently, induce GEM resistance. This suggests that GEM treatment itself might induce GEM resistance, even in clinical situations. However, it will be important to investigate whether knockdown of the drug resistance-associated genes, RRM and MPR1, in MIA-G cells negates the resistance-induced changes to energy metabolism and the redox system. It will also be similarly important to examine the effects of knocking down dCK in MIA-P cells. Our results suggest that effective intervention to maintain normal energy metabolism in cancer cells is necessary to prevent the acquisition of GEM resistance or to suppress resistance.

## 4. Materials and Methods

### 4.1. Cell Lines

The human PDAC cell line MIA-PaCa-2 (MIA-P cells) was purchased from Dainihon Pharmaceutical Co. (Tokyo, Japan) [61]. Aspc1, Capan2, Panc1, and Panc2 human PDAC cell lines were provided from the ATCC. MIA-P cells were cultured in Dulbecco’s Modified Eagle Medium (DMEM) (Wako Pure Chemical Co., Ltd., Osaka, Japan) supplemented with 10% fetal bovine serum (Sigma-Aldrich Inc., St. Louis, MO, USA) at 37 °C in 5% CO_2_. The therapeutic blood concentration of GEM was up to 10 to 30 µg/mL after a one-shot venous injection [62]. To establish a GEM-resistant cell line, PDAC cells were continuously treated with therapeutic-dose GEM (0.1 nM) for 50 passages. The resistance of the MIA-G cell line to GEM was confirmed using cell growth, apoptosis, invasion, and tumor formation assays.

### 4.2. Cell Growth and Apoptosis Assays

Cell growth was assessed using the tetrazolium (MTT) dye assay (Wako) [63], and the 3-(4,5-dimethylthiazol-2-yl)-5-(3-carboxymethoxyphenyl)-2-(4-sulfophenyl)-2H-tetrazolium (MTS) assay (Promega Corporation, Madison, WI, USA) [64], as previously described. Apoptotic cell number was assessed by observing 1000 cells with Hoechst 33342 dye staining (Life Technologies, Carlsbad, CA, USA) [65].

### 4.3. Animals

BALB/c nude mice (male, 4 weeks old) were purchased from Japan SLC (Shizuoka, Japan). The experiments were performed in compliance with the institutional guidelines approved by the Committee for Animal Experimentation of Nara Medical University, which conformed to the current regulations and standards of the Ministry of Health, Labor, and Welfare (approval no.160929). For tumor formation assays, 1 × 10^7^ MIA-P or MIA-G cells were inoculated subcutaneously into the scapular tissues of three nude mice in each group. GEM (10 mg/kg body weight) was injected into the peritoneal cavity on day one and the tumor size was monitored weekly.

### 4.4. Invasion Assay

Invasive ability was assessed using a modified Boyden chamber assay, as described previously [63]. After incubation for 24 h at 37 °C, filters were carefully removed from the inserts. The remaining cells were stained with hematoxylin for 10 min and mounted on microscope slides. The number of stained cells was counted on a light microscope at 100× magnification. The average number of cells per insert was quantified as the invasive ability.

### 4.5. Reagents

GEM and N-acetyl-L-cysteine (NAC) were purchased from Sigma-Aldrich. 5-Fluorouracil (5-FU), cisplatin (CDDP), vitamin C, and rotenone were purchased from Wako.

### 4.6. Immunoblot Analysis

Whole-cell lysates were prepared as previously described [66]. Protein lysates (25 μg) were separated on 12.5% sodium dodecyl sulfate-polyacrylamide gels, followed by being electrotransferred onto a nitrocellulose filter. The membranes were incubated with primary antibodies, and then with peroxidase-conjugated IgG antibodies (Agilent Technologies, Santa Clara, CA, USA). Immune complexes were detected using an ECL Western blot detection system (Amersham, Aylesbury, UK). The following primary antibodies were used, at a working dilution of 1:1000, for immunoblot analyses: antibodies against CD44 (c-9960), CD47 (sc-7059), nucleostemin (NS) (sc-166460), MDR (sc-13131), RELA (sc-372), β-actin (sc-47778) (Santa Cruz Biotechnology Inc., Dallas, TX, USA), E-cadherin (Abcam, Cambridge, MA, USA; ab1416), snail (#3879), and phospho-Akt (Ser473) (#4060) (Cell Signaling Technology Japan, Tokyo, Japan).

### 4.7. Enzyme-Linked Immunosorbent Assay (ELISA)

An ELISA kit (human mtTFA ELISA Kit; Abcam, Cambridge, MA, USA) was used to measure the concentration of mitochondrial transcription factor A. Glutathione peroxidase (GPx) activity was measured using a glutathione peroxidase assay kit (Japan Institute for the Control of Aging, NIKKEN SEIL Co., Ltd., Tokyo, Japan). The assay was performed according to the manufacturer’s instructions, using whole-cell lysates.

### 4.8. Reverse Transcription-Polymerase Chain Reaction (RT−PCR)

To assess the expression of drug resistance-related genes, RT-PCR was performed using cDNA synthesized from 1.0 µg of total RNA extracted with an RNeasy kit (Qiagen, Germantown, MD, USA). The primer sets used are listed in Table 1. Primers for hENT1, dCK, RRM1, and RRM2 were based on a previously described sequence [50]. The primer sets were synthesized by Sigma Genosys (Ishikari, Japan). The PCR products were electrophoresed on a 2% agarose gel and stained with ethidium bromide, then photographed under UV light.

### 4.9. Flux Analysis

To measure the oxygen consumption rate (OCR), 1.5 × 10^4^ cells were cultured in a Seahorse XFp Cell Culture Miniplate (Agilent Technologies) for 12 h at 37 °C, with or without GEM. The medium contained Seahorse XF DMEM (pH 7.4) containing pyruvate (1 mM), glutamine (2 mM), and glucose (10 mM). The Mito Stress Test was performed using the Seahorse XFp analyzer, in which cells, oligomycin (2.0 μM), carbonyl cyanide-4-(trifluoromethoxy) phenylhydrazone (FCCP) (1.0 μM), and rotenone (0.5 μM) were added sequentially. To assess L-glutamine uptake, an L-glutamine oxidation assay was performed using 4 mM L-glutamine [67]. For each assay, the total protein concentration was used for normalization. Whole-cell lysates were extracted using M-PER Mammalian Protein Extraction Reagent (Thermo Scientific, Rockford, IL, USA).

### 4.10. Mitochondrial Imaging

To evaluate mitochondrial status after GEM treatment, 2.0 × 10^4^ cells were seeded in 12-well plates with or without GEM (0.1 μM), and each assay was performed after 72 h of incubation. The mitochondrial volume was assessed using MitoGreen (100 nM, for 15 min at 37 °C, PromoCell GmbH, Heidelberg, Germany). Mitochondrial potential was examined using tetramethylrhodamine ethyl ester (TMRE) (200 nM for 30 min at 37 °C, Sigma-Aldrich). Mitochondrial ROS levels were examined using dihydrorhodamine 123 (DHR123; 10 μM, 20 min at 37 °C; Sigma-Aldrich). Ethidium bromide (EtBr) was used to detect mtDNA and cytosolic EtBr accumulation was measured. Before imaging, cells were washed with Hank’s balanced salt solution. Fluorescence was measured using an all-in-one fluorescence microscope (BZ-X710; KEYENCE Co., Itasca, IL, USA). We used the NIH ImageJ software (version 1.52, Bethesda, MD, USA) to analyze the median fluorescence intensity per cell.

### 4.11. Patients

We obtained frozen tissue samples from 7 patients with PDAC, which were diagnosed at the Department of Molecular Pathology, Nara Medical University, during 2012–2019. All patients received at least 6 courses of postoperative chemotherapy with GEM monotherapy. Three patients were considered GEM sensitive because they had either a complete response, partial response, or stable disease at the end of the 6 courses. The other four patients were classified as progressive disease and GEM resistant. As written informed consent was not obtained from the patients for their participation in the present study, all identifying information was removed from patient samples prior to their analysis to ensure strict privacy protection (unlinkable anonymization). All procedures were performed in accordance with the Ethical Guidelines for Human Genome/Gene Research enacted by the Japanese Government and with the approval of the Ethics Committee of Nara Medical University (approval number: 937, 1 April 2012).

### 4.12. Statistical Analysis

Statistical significance was calculated using a two-tailed Fisher’s exact test, one-way ANOVA, and Student’s *t*-test using the InStat software (GraphPad, LOS Angeles, CA, USA). Statistical significance was set at a two-sided *p*-value < 0.05.

## Figures and Tables

**Table 1:** Primer sequences.

Gene	Gene Bank ID	Forward	Reverse
*β-actin*	BC002409.2	GGACTTCGAGCAAGAGATGG	AGCACTGTGTTGGCGTACAG
*hENT*	Yoneyama et al. [50]	TGTTTCCAGCCGTGACT	CAGGCCACATGAATACAG
*dCK*	Yoneyama et al. [50]	TGCAGGGAAGTCAACATT	TCCCACCATTTTTCTGAG
*RRM1*	Yoneyama et al. [50]	CGCTAGAGCGGTCTTATTTGTT	TTGCTGCATCAATGTCTTCTTT
*RRM2*	Yoneyama et al. [50]	CCCGCTGTTTCTATGGCTTC	CCCAGTCTGCCTTCTTCTTG
*MRP1*	L05628.1	TGAAGG ACTTCGTGTCAGCC	GTCCATGAT GGTGTTGAGCC
*TFAM*	EU279428.1	CCCCCACAAACCCCATTACTAAACCCA	TTTCATCATGCGGAGATGTTGGATGG
*MYCC*	V00568.1	TTCGGGTAGTGGAAAACCAG	CAGCAGCTCGAATTTCTTCC

## Data Availability

Not applicable.

## Abbreviations

PDAC—pancreatic ductal adenocarcinoma; MIA-P—MIA-PaCa-2; MIA-G—MIA-PaCa-GEM; GEM—gemcitabine; 5-FU—5-fluorouracil; CDDP—Cisplatin; OXPHOS—oxidative phosphorylation; MMP—mitochondrial membrane potential; mtROS—mitochondrial reactive oxygen species; TFAM—mitochondrial transcription factor A; mtDNA—mitochondrial DNA; TMRE—tetramethylrhodamine ethyl ester; DHR—dihydrorhodamine, OCR—oxygen consumption rate; ECAR—extracellular acidification; FCCP—4-trifluoro-methoxy-phenyl-hydrazone; GSH—glutathione-SH; GSSG—glutathione disulfide; GPx-1—glutathione peroxidase; NAC—N-acetyl-L-cysteine; EMT—epithelial–mesenchymal transition; AKT—AKT serine/threonine kinase; NS—nucleostemin; MDR—multidrug resistance protein 1; RELA—nuclear factor kappa-light-chain-enhancer of activated B cells 3; ACTB—β-actin; hENT—human equilibrative nucleoside transporter 1; DCK—deoxycytidine kinase; RRM—ribonucleotide reductase; MRP1—multidrug resistance-associated protein 1.
